# Sleepless in Society: Introducing the Concept of Public Sleep

**DOI:** 10.3390/clockssleep8020018

**Published:** 2026-04-09

**Authors:** Tony J. Cunningham, Shengzi Zeng, Seo Ho Song

**Affiliations:** 1 Center for Sleep and Cognition, Department of Psychiatry, Beth Israel Deaconess Medical Center, 330 Brookline Ave, Boston, MA 02215, USA; 2 Division of Sleep Medicine, Harvard Medical School, Boston, MA 02215, USA

**Keywords:** public sleep, public mood, daylight savings time, social rhythms, collective trauma

## Abstract

Major social, cultural, and sociopolitical events routinely disrupt daily life, yet their effects on sleep are rarely conceptualized at the population level beyond anecdotal sharing. The purpose of this *Opinion* piece is to initiate a preliminary discussion of “public sleep” as a novel construct describing systematic, event-related changes in sleep timing, duration, and quality that emerge coherently within communities in response to shared social experiences. Drawing on similarities with the well-established concept of public mood, we posit that sleep can be shaped by social environments in which shared attention, emotional climate, and coordinated schedules exert systematic influence. In support of this claim, we describe preliminary evidence from diverse domains demonstrating population-level sleep disruption following major events, including the transition to Daylight Saving Time, national elections, prolonged crises such as the COVID-19 pandemic and armed conflicts, and highly salient cultural activities such as major sporting events. These reports from disparate fields provide an initial indication that public sleep disruptions can be acute or prolonged, geographically localized or global, and may be shaped by the duration, emotional intensity, and perceived importance of the associated event. We further highlight the potential public health, safety, social, and economic consequences of collective sleep loss, underscoring its relevance beyond individual well-being. Finally, we outline key directions for future research, emphasizing the need for systematic reviews, mechanistic studies, longitudinal designs, and policy-relevant recommendations. Recognizing public sleep as a measurable population phenomenon would provide a foundation for anticipating, monitoring, and mitigating sleep disruption during periods of collective strain, with implications for both individual health and societal resilience.

## 1. Introduction

On 27 October 2018, Red Sox fans watched their team enter the bottom of the 18th inning against the Los Angeles Dodgers in what had already become the longest World Series game in history [1]. The game had stretched on for 7 h and 20 min, long past any reasonable bedtime, finally ending at 3:30 a.m. on the East Coast. By the time the final out was recorded, sleep had been postponed, alarms were only a few hours away, and it would be reasonable to assume that New England coffee shops went on to have a banner day. For thousands of fans across the northeast, this was not just a memorable sporting moment but a shared night of curtailed sleep. Experiences like this highlight how major public events can meaningfully disrupt sleep at a community level. This kind of collective sleep loss, scaled across fandoms, cities, or even nations, invites a broader question about how shared social experiences shape our sleep.

## 2. Defining ‘Public Sleep’

While most can likely relate to these types of anecdotal events, mounting evidence suggests that these experiences may be more frequent, more ubiquitous, and potentially more problematic than we have previously considered. In light of this growing body of empirical support, we propose our idea for the construct of “**Public Sleep**” as systematic, event-related changes in sleep timing, duration, or quality that emerge at the group level because individuals, as members of a shared social community, harmonize their sleep-related behaviors and physiology leading up to and in the wake of emotionally salient public events. These changes are not random fluctuations or the aggregation of idiosyncratic sleep disturbances, but coherent shifts that arise in response to shared experiences such as major sporting events, elections, natural disasters, pandemics, war, or other socially meaningful disruptions. Public sleep is therefore inherently contextual and collective, characterized by its sensitivity to the duration, breadth, emotional intensity, and perceived severity and importance of an event, as well as by its potential to propagate across communities through social identity and media exposure. Critically, public sleep can manifest as measurable population-level changes, including shifts in mean sleep duration (e.g., ≥30 min) [2,3,4], alterations in sleep timing (e.g., bedtime and wake time changes ≥ 30 min) [5,6], changes in variability and regularity (including synchronization across individuals) [6,7], significant changes in insomnia symptoms [8,9], and changes in the proportion of individuals crossing clinically meaningful thresholds (e.g., short sleep duration of <6 h) [8,10]. These patterns may vary depending on whether events constrain, delay, relax, or otherwise restructure daily routines and social demands.

This concept closely parallels the established construct of ‘public mood’, defined as broad changes in affective state individuals experience due to their membership in a political or social community [11,12]. Public mood has been shown to be driven by events that are perceived as both positive and negative, and demonstrates that collective events such as elections, national tragedies, and major sporting events reliably shift affect across communities, influencing judgment, well-being, and behavior beyond individual-level appraisals [12,13,14,15]. Multiple studies have demonstrated that the outcome of sporting events can not only impact the mood and perception of general life satisfaction among fans, but it can also influence suicidality and homicidality rates as well [13,16,17,18]. Major sociopolitical events, like the Brexit referendum and recent US elections, have also been shown to affect measures of emotional well-being, such as stress, anxiety, and depression [2,4,12,19,20]. Parasocial relationships lead to impacts on mood and expressions of grief following the death of celebrities [21,22,23]. Additionally, a flood of research during the COVID-19 pandemic demonstrated that these events do not need to be short-term or acute in nature. Rather, the magnitude and duration of an event may lead to a more pervasive, sustained impact [24,25,26,27,28,29]. Importantly, public mood is not simply the average of private emotions, but a social state shaped by shared attention, collective memory, and social identity [30], which can be further enhanced via news and social media outlets [21,23,31,32]. We posit that public sleep is a direct behavioral and physiological analogue to public mood. When events reorganize attention, arousal, time use, and emotional bandwidth at a societal level, sleep, like affect, is affected. Importantly, this distinction implies that public sleep, like public mood, represents emergent, socially patterned phenomena rather than simple aggregations of independent individual responses. Rather than isolated, unpredictable reactions, the magnitude and direction of these effects are shaped by factors such as degree of identification with the affected community, perceived severity and duration of the event, and its predictability, leading to coordinated and sometimes anticipatory shifts in behavior across populations. In this way, public sleep reflects structured, partially predictable changes in sleep behavior that arise from shared social processes, rather than merely the sum of unrelated individual sleep changes, and this predictability may offer opportunities for targeted intervention.

## 3. Supporting Evidence for Public Sleep

One of the most pervasive and well-studied examples of population-level sleep disruption occurs each spring with the transition to Daylight Saving Time (DST). Unlike many other societal events, the “spring forward” shift affects nearly the entire population in each participating country simultaneously and is externally imposed rather than discretionary, making it a natural experiment in public sleep disruption. Multiple studies demonstrate that this transition is associated with an immediate reduction in sleep duration, with estimates suggesting an average loss of approximately 30 to 40 min of sleep on the first night following the shift [33,34]. Importantly, the effects are not limited to a single night. Experimental and observational work indicates that it takes several days for sleep timing to realign to the new clock time [35,36,37]. While some studies indicate a timeline of five to seven days for circadian and sleep timing adjustments to occur [35,36], mounting evidence has begun to indicate that it may take considerably longer, especially in short sleepers and individuals with late chronotypes [37,38]. These findings likely underestimate the full impact of the transition, as they do not fully account for circadian misalignment driven by altered light exposure, which may further degrade sleep quality and alertness during the adjustment period [35]. Together, the DST literature provides a clear demonstration of public sleep as a coherent, population-wide phenomenon characterized by predictable changes in sleep duration, timing, and recovery.

Elections provide a complementary example of public sleep disruption that differs from Daylight Saving Time in both mechanism and emotional valence. Rather than being driven by an externally imposed schedule change, election-related sleep disruption appears to arise from heightened emotional arousal, uncertainty, and prolonged media engagement [2,3,39,40]. A 2019 study aggregated 15 million nights of sleep using sleep-monitoring smartphone apps surrounding the UK Brexit referendum and the 2016 US presidential election and found a significant reduction in sleep duration for UK and US residents, respectively [39]. Using daily assessments surrounding the 2020 U.S. elections, our group documented significant reductions in sleep duration and quality on election night [2]. We replicated these core findings during the 2024 US election, again demonstrating acute sleep loss on election night followed by compensatory recovery sleep, alongside prolonged disruptions in mood and psychological well-being [4]. These studies also revealed two tantalizing findings deserving further exploration and replication. First, results from both our group and Anýž et al., indicated that the impacts of recent US elections on sleep surprisingly extended beyond US borders [2,39]. This suggests that as globalization increases, national interests become more interconnected, and information flow increases through news and social media, distinctions between communities may increasingly blur, allowing major events in one country to produce broader, potentially global, changes in mood and sleep. Second, the temporal profile of the 2024 US election effects differed from those observed in 2020, with stress and affective disturbances taking longer to return to baseline, underscoring how the duration and perceived severity of sociopolitical events may modulate the persistence of both public mood and public sleep disruptions [2,4]. These initial findings illustrate how emotionally charged political events can reliably affect sleep behavior among various populations even in the absence of formal changes to social schedules.

Given the mounting evidence that stress surrounding democratic elections can influence sleep behavior, it is likely unsurprising that there are also significant sleep health consequences when diplomacy breaks down. A 2023 assessment of Ukrainian residents found that in the year after Russia’s initial invasion, 39.4% of the respondents reported a short sleep duration average (≤6 h) and 38.5% met criteria for insomnia—more than 3x the typical 10–12.4% population prevalence [8,41,42]. A study of 1458 Palestinian adults living in the Gaza strip in early 2022 revealed that over half reported poor sleep quality, with the prevalence of short sleep duration and excessive daytime sleepiness being 26.4% and 43.6%, respectively [10]. A cross-sectional study of Lebanese university students living in Lebanon during the 2023–2024 conflicts indicated that 73.5% had poor sleep quality as measured by the Pittsburgh Sleep Quality Index (PSQI) [43] and that individuals were 19.76 times more likely to suffer from poor sleep if they experienced direct war exposure [44]. While events like DST and elections come with clear and expected timelines, these studies underscore the methodological challenges inherent in studying public sleep disruption in certain settings, such as armed conflict, where complex historical contexts, ambiguous onset and resolution of events, sustained uncertainty, and fluctuating exposure levels complicate the identification of clear boundary conditions, temporal anchors, and causal inferences. These studies also revealed important potential moderators in need of further investigation, including biological sex, age, psychiatric history, war exposure and experiences of trauma, and loneliness.

Other large-scale public events further underscore the breadth of contexts in which public sleep emerges. During the COVID-19 pandemic, population-level studies using surveys, smartphone applications, and wearable devices consistently documented widespread shifts in sleep timing and duration [5,6,7,45,46]. In contrast to the acute sleep loss seen with clock changes and elections, population-level pandemic-related changes were typically characterized by delayed sleep timing, modest increases in sleep duration, and reduced weekday–weekend variability, likely reflecting the significant alteration in work schedules and social expectations [5,6,46,47,48,49]. At the same time, many individuals reported declines in sleep quality, highlighting how extended stressors can simultaneously increase sleep opportunity while degrading subjective sleep health [9,45]. During the height of the COVID-19 pandemic, the loss of social rhythms due to distancing and lockdown policies may have further exacerbated sleep, circadian, and mood concerns, especially among those with pre-existing psychiatric conditions [50,51]. Research such as this highlights both potential high-risk groups and possible interventions to mitigate the impact under similar circumstances in the future, as discussed in more detail below.

In contrast to the prolonged and diffuse disruptions observed during the COVID-19 pandemic, other types of natural disasters often represent more acute, high-intensity events that can produce more immediate disturbances in sleep [52]. Similarly to living in a community experiencing conflict, however, natural disasters typically involve substantial uncertainty and collective trauma that may prolong its impact, extending it through the recovery and rebuilding period following the event. In a study of L’Aquila, Italy residents before and after a 6.3 magnitude earthquake, researchers found significant deterioration in sleep quality in the wake of the disaster, particularly in the elderly [53]. Moreover, proximity to the epicenter of the earthquake predicted sleep disturbances and disruptive nocturnal behaviors. A systematic review of wildfire survivors has revealed that 63–72.5% of the members of affected communities report insomnia and 33.3–46.5% report nightmares in the months following the event, with potential links of sleep disruption to the severity and proximity of the fires [54]. Following the 2008 Wenchuan earthquake in China, nearly half of a group of 1573 adolescents reported sleeping less than 7 h per night on average, with 27.7% reporting difficulties initiating sleep and 22.6% reporting poor sleep quality [55]. A notable challenge in studying sleep during natural disasters as a means of understanding public sleep is their inherently unpredictable onset, which, coupled with the immediate need to prioritize safety, shelter, and other critical survival-related demands, often limits the feasibility of capturing real-time or pre-event sleep data and complicates efforts to determine the temporal dynamics of sleep disruption. As such, much of the existing literature in this area has relied on retrospective assessments, and while limited, available evidence suggests that sleep disturbances following natural disasters can persist for months to years after the initial event [53,55,56,57]. Importantly, these prolonged sleep disruptions have been linked to the development and maintenance of PTSD and other psychiatric symptoms, underscoring the potential value of understanding public sleep in the aftermath of such events as a means of informing interventions aimed at enhancing community resilience [52,55,56,57,58,59,60,61].

Finally, it is important to note that more transient but highly salient cultural events, such as holidays, religious celebrations, and major sporting events like the Super Bowl and World Series, offer additional illustrations of public sleep in action. New Year’s Eve has been linked to a 13.8% reduction in sleep consistency and an 89 min delay in sleep onset [62]. Observance of Ramadan has been associated with an average sleep duration decrease of approximately one hour across the month as compared to the rest of the year [63,64]. National surveys conducted by the American Academy of Sleep Medicine (AASM) indicate that substantial proportions of the population report increased tiredness following these events, particularly on the mornings after late-night games [65,66]. Although these sporting events affect smaller segments of the population compared to elections or pandemics, they nonetheless demonstrate how shared attention to a common event can produce measurable, collective shifts in sleep. Further, while most Red Sox and Dodger fans were lucky to be able to rest on a Saturday following their record breaking World Series game in 2018, East Coast Dodger and Astro fans were not as fortunate in 2017 when Game 5 went to 1:37 am on a Monday morning, setting them up to enter their work week with significantly reduced sleep and potentially serious consequences. Taken together, these diverse examples support the central premise that socially meaningful events, whether brief or prolonged, can systematically shape sleep behavior across communities in ways that are predictable, measurable, and consequential.

## 4. Why Public Sleep Matters

Daylight Saving Time again acts as an intriguing and compelling natural experiment with significant empirical data illustrating the real-world consequences of public sleep disruption [33,34,35,36,37,67]. In a seminal position statement, the AASM concluded that cumulative evidence confirms that the spring transition incurs significant public health and safety risks, including heightened risks of cardiovascular events, mood disorders, and motor vehicle accidents [68,69]. Systematic reviews have demonstrated that the spring transition is associated with immediate reductions in sleep duration on the first night as well as cumulative impairments across the subsequent week as increased sleep fragmentation and sleep latency compound the initial loss [35,37]. Further, evidence indicates that while most eventually adjust after a week or two, some individuals fail to realign their circadian clock for the duration of the daylight saving period [35,36,38] potentially leading to more pervasive, long-term impacts with more severe, detrimental outcomes on emotional and physical well-being [70].

The functional ramifications of such sleep disruptions are substantial. Even modest sleep restriction compromises cognitive and motor function equivalent to legally significant levels of alcohol intoxication [71,72,73,74,75]. Specifically, sustaining wakefulness for 17 to 19 h without rest equivocates to functional decline from blood alcohol concentrations of 0.05% as manifested by significantly compromised arousals, stimulus response, and executive function [72,74,75]. Sleep deprivation exceeding 24 h yields functional impairment that is expected from blood alcohol concentrations of 0.1%, exceeding legal intoxication thresholds in the USA. These suggest that widespread public sleep disruption following major events increases the risk of major and fatal accidents at the population level [71,72,74,75].

In addition to deterioration of cognitive abilities, a substantial body of literature further indicates that chronic sleep disruption is closely intertwined with adverse mental health outcomes across a wide range of contexts, including increased risk for depression, anxiety, and impaired emotional regulation [13,52,59,76,77,78]. While these types of long-term outcomes are almost certainly linked to the affect, severity and predictability of the event, in the aftermath of large-scale stressors such as natural disasters and terrorist attacks, including work following events like 9/11, sleep disturbances have been consistently associated with the onset and persistence of posttraumatic stress symptoms and broader psychological distress, often persisting well beyond the acute phase of the event [52,55,56,61,79]. Importantly, emerging evidence also suggests that sleep may serve as a modifiable pathway through which resilience operates, highlighting the potential for targeted interventions aimed at preserving or restoring sleep in the wake of major shared stressors to not only improve individual well-being but also to strengthen mental health and resilience at the community level [59,80,81].

Beyond safety and emotional well-being, public sleep disruption may erode the social fabric itself. Even a single night of sleep loss triggers withdrawal from prosocial helping behavior, associated with deactivation of key nodes within brain networks linked to social cognition [82]. Population-level analyses from Simon and colleagues’ (2022) noted a 10% reduction in charitable donations following the one-hour sleep loss imposed by springtime daylight saving transition. In the same report, experimental manipulation of sleep affected willingness to engage in helping behavior. Another study found that insufficient sleep is associated with reduced engagement in civic duties, voting, and prosocial behaviors [83]. Sleep, therefore, fuels human prosociality; collective disruption of sleep diminishes the cooperative behaviors that underscore community functioning, which may further exacerbate and prolong the effects on public sleep and mood.

The potential economic costs of population-level sleep disruption are no less staggering. RAND Europe estimates that sleep insufficiencies cost the US economy close to $411 billion annually—or 2.28% of its GDP—through a combination of reduced productivity, more frequent absenteeism, and increased mortality risk [84]. Notably, an estimated 1.2 million working days are lost each year due to sleep deprivation among the US workforce alone [84]. Hypothetically, restoring an hour of sleep in individuals sleeping less than six hours could recover $266 billion to the US economy [84]. When public events systematically curtail sleep across communities, multiplicative accrual of individual sleep losses substantiate real and measurable economic consequences.

## 5. The Future of Public Sleep Research

We believe that there already exists substantial evidence across different research domains to support the construct of public sleep as we have described, however much remains unknown. For instance, while the literature provides convincing evidence that public events alter sleep duration, timing, and regularity at the population level, the depth and architecture of these disruptions remain poorly understood. Such gaps in our knowledge are critical as they are essential for designing precise loci for interventions. One notable limitation of the work thus far is that most studies to date have relied on self-report measures or consumer-grade wearable hardware, which inform sleep timing and subjective quality and estimate sleep duration but offer limited insight characterizing sleep physiology vis-à-vis sleep stage compositions, EEG spectra, or other markers of sleep quality and restoration [5,6,45,85]. Future research employing high-precision polysomnography, research-grade actigraphy, and validated objective sleep assessments in ecologically valid settings is crucial for decoding the physiological features of public sleep disruption. Such works will reveal whether event-related sleep changes preferentially impact specific sleep stages (e.g., REM sleep, slow wave sleep, etc.) with implications for emotional regulation, memory consolidation, and next-day functioning.

Our prior work showed that changes in sleep during the 2020 US Presidential Election significantly associated with concurrent shifts in mood and affective state [2]. However, the directionality and mechanisms underlying this relationship are yet to be fully elucidated. Do emotionally charged events first disrupt sleep prior to destabilizing affect? Does heightened affective arousal impair sleep initiation and/or maintenance? Or do bidirectional feedback loops amplify both disturbances simultaneously? Longitudinal designs with robust repeated measures—ideally recording sleep and mood simultaneously at multiple time points—before, during, and after major public events, will help parse these temporal dynamics. Ecological momentary assessment (EMA), which captures real-time or near real-time reports of sleep, mood, and contextual factors in naturalistic settings, represents a complementary approach that can further enhance temporal resolution and reduce recall bias in studying these dynamic processes. Dissecting the interplay between public sleep and public mood will be critical to reveal targets of intervention where addressing one domain could bilaterally mitigate disruptions in the other.

A related challenge in this area is that many of the most consequential events that influence public sleep, including armed conflict and natural disasters, are inherently unpredictable, limiting the ability to prospectively capture baseline data and precisely define temporal dynamics. One potential approach to advancing this field is to initially focus on predictable, recurrent events such as elections, daylight saving time transitions, holidays, and major sporting events, which provide natural opportunities for structured, prospective study designs. If these approaches prove fruitful, it may then be worthwhile to extend such methodologies to regions at elevated risk for conflict or environmental disasters through the implementation of ongoing or periodic monitoring efforts. Establishing such infrastructure could enable the capture of high-resolution sleep and behavioral data before, during, and after unexpected events, thereby substantially enhancing our ability to characterize and respond to public sleep disruption in real-world settings.

Another challenge in developing a full understanding of public sleep is the substantial diversity of events and community sizes that may be relevant to its expression, ranging from households, classrooms, and workplaces to cities, regions, and entire nations. Similarly to how different forms of trauma can give rise to different constellations of posttraumatic symptoms [86,87], different community events likely produce distinct impacts on sleep and downstream effects on mood, cognition, and functioning. These effects may arise through different mechanisms, such as social synchronization, emotional contagion via media, and disruption of social rhythms and zeitgebers, and may also vary according to moderators such as age, sex, gender, chronotype, psychiatric history, directness of exposure, psychiatric history, loneliness, socioeconomic status, and the predictability, duration, and perceived significance of the event itself. This heterogeneity underscores the need for creative and strategic investigation of the construct. For instance, while a single systematic review or meta-analysis spanning all potentially relevant contexts may not be practical, the field could make substantial progress by first synthesizing findings within related clusters of events, such as the recurring and predictable disruptions highlighted above, before expanding to more complex and less predictable contexts (e.g., planned vs. unplanned; acute vs. chronic; political vs. natural vs. cultural, etc.). Another approach could be to move beyond event type and instead characterize events along shared dimensions—such as predictability, duration, emotional valence, and degree of social synchronization—allowing for comparisons across otherwise heterogeneous contexts.

Given these challenges, previous work on public mood provides a valuable conceptual and methodological template for advancing the study of public sleep, particularly in its use of population-level metrics, repeated sampling, and event-based analyses. It is worth explicitly noting that while we believe these concepts are related, we do not view them as interchangeable. For instance, while public sleep may exhibit similar patterns of disruption or change across a majority of individuals exposed to the same event, public mood responses have been shown to diverge substantially depending on the outcome [2,4]. For example, while an election or sporting event may produce widespread sleep loss, emotional responses may differ drastically depending on the outcome for your preferred candidate or team. Indeed, some work suggests that mental health outcomes can improve following the resolution of certain stress-inducing events [13], further underscoring the importance of disentangling the shared and distinct dynamics of public sleep and public mood.

As work in this space continues to progress and become more rigorously characterized, there will be a growing need to move beyond descriptive accounts toward testable models of how public sleep emerges and evolves. At present, however, we view any such framework as necessarily provisional, given the complexity and heterogeneity of events and populations involved. One plausible, simplified model is that shared public events first alter collective attention and emotional climate (e.g., public mood), which then influence sleep through multiple interacting pathways, including social synchronization of behavior, emotional contagion via media exposure, and disruption of social rhythms and zeitgebers. These pathways likely operate in parallel and may be differentially engaged depending on characteristics of the event (e.g., predictability, duration, severity) and individual or group-level moderators (e.g., age, sex, psychiatric history, degree of community identification, and proximity of exposure). Importantly, feedback loops may further amplify or sustain these effects, especially as events transition from acute to chronic, as sleep disruption itself can influence mood, behavior, and social engagement. We present this as one potential starting point rather than a definitive framework, with the expectation that future empirical work will be needed to refine, expand, and potentially revise these relationships and their contributions. Importantly, the ultimate goal of defining a rigorous framework of public sleep is to invite consideration and opportunities to pioneer sleep-based interventions at individual and societal levels through a diversity of tools ranging from established treatment plans to policy changes. At the individual level, evidence-based methods for managing acute sleep disruption—e.g., stimulus control, sleep restriction, relaxation training, CBTi, etc.—could be disseminated in anticipation of predictable high-impact events such as elections, major sporting occasions, or scheduled clock changes [68]. Public health education can raise awareness that collective events may impair sleep and offer practical guidance for risk reduction, such as maintaining social rhythms and sleep regularity. At the policy level, the growing scientific consensus against biannual clock changes illustrates that public sleep research can inform consequential decisions [68,69]. Subsequent work should examine population-level impacts of scheduling major events at times that minimally impact sleep. Recognizing public sleep as a measurable construct opens possibilities for monitoring population sleep health in real time and engineering early warning systems for periods of anticipated, elevated risk.

### Concluding Remarks

In this paper, we introduce the idea of **public sleep** as a population-level phenomenon in which the duration, timing, and quality of sleep shift coherently within a community in response to shared social experiences. Rather than reflecting isolated or idiosyncratic disturbances, we believe that public sleep captures systematic changes that arise because individual members of larger communities attune their sleep in response to salient public events. Drawing parallels with the well-established construct of public mood, we argue that sleep is sensitive to collective attention, emotional climate, and shared constraints on time and behavior. Empirical evidence supports drawing a parallel between the concepts of public mood and public sleep, with population-level sleep and mood changes observed following elections in multiple countries, during prolonged collective stressors such as the COVID-19 pandemic, and in response to highly salient cultural events. Together, these findings challenge the notion that sleep is solely a private behavior between an individual or couple and instead position it as a socially embedded process shaped by forces that operate beyond the bedroom.

Recognizing public sleep as a distinct and consequential construct carries important implications for science, public health, and policy. The evidence reviewed here indicates that collective sleep disruption is not merely inconvenient, but may be meaningfully linked to health, safety, social functioning, and economic productivity. As societies become increasingly interconnected and exposed to rapid information flow, the potential for widespread sleep disruption is likely to grow rather than diminish. Thus, we call for a coordinated research agenda that treats public sleep as a measurable signal, worthy of the same empirical rigor that has advanced the study of public mood. We hope that this introductory description of public sleep serves as a catalyst for further discussion, refinement, and empirical investigation of this concept within the field. By systematically studying how, when, and for whom public events disrupt sleep, the field can move toward anticipatory interventions, informed policy decisions, and strategies that protect sleep health during periods of collective strain. In doing so, public sleep research has the potential not only to improve individual well-being, but also to strengthen the resilience of communities themselves.

## Data Availability

No new data were created or analyzed in this study. Data sharing is not applicable to this article.
